# Extraordinary Thermal Stability and Sinter Resistance of Sub-2 nm Platinum Nanoparticles Anchored to a Carbon Support by Selenium

**DOI:** 10.1021/acs.nanolett.3c04601

**Published:** 2024-01-16

**Authors:** Zitao Chen, Haoyan Cheng, Zhenming Cao, Jiawei Zhu, Thomas Blum, Qinyuan Zhang, Miaofang Chi, Younan Xia

**Affiliations:** † The Wallace H. Coulter Department of Biomedical Engineering, Georgia Institute of Technology and Emory University, Atlanta, Georgia 30332, United States; ‡ State Key Laboratory of Luminescent Materials and Devices, South China University of Technology, Guangzhou 510641, China; § Center for Nanophase Materials Sciences, Oak Ridge National Laboratory, Oak Ridge, Tennessee 37831, United States; ∥ School of Chemistry and Biochemistry, Georgia Institute of Technology, Atlanta, Georgia 30332, United States

**Keywords:** electron microscopy, *in situ* heating, Pt nanoparticles, sinter resistance, thermal stability

## Abstract

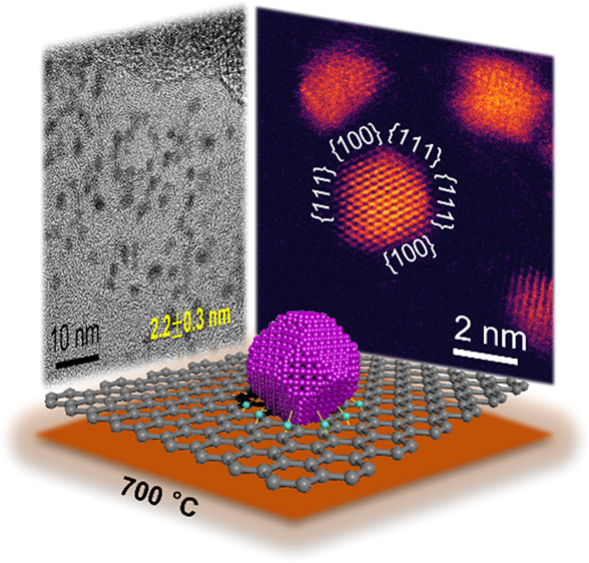

Nanoparticle sintering has long been a major challenge in developing catalytic systems for use at elevated temperatures. Here we report an *in situ* electron microscopy study of the extraordinary sinter resistance of a catalytic system comprised of sub-2 nm Pt nanoparticles on a Se-decorated carbon support. When heated to 700 °C, the average size of the Pt nanoparticles only increased from 1.6 to 2.2 nm, while the crystal structure, together with the {111} and {100} facets, of the Pt nanoparticles was well retained. Our electron microscopy analyses suggested that the superior resistance against sintering originated from the Pt–Se interaction. Confirmed by energy-dispersive X-ray elemental mapping and electron energy loss spectra, the Se atoms surrounding the Pt nanoparticles could survive the heating. This work not only offers an understanding of the physics behind the thermal behavior of this catalytic material but also sheds light on the future development of sinter-resistant catalytic systems.

Carbon-supported Pt nanoparticles are important catalysts for a variety of reactions, including those vital to the operation of proton-exchange membrane fuel cells.^1^ The performance of this catalytic system critically depends on the size of the nanoparticles. In general, the nanoparticles should be made and kept as small as possible to ensure a large specific surface area and thus a high mass activity.^2,3^ However, smaller nanoparticles are more susceptible to sintering (i.e., growth into larger particles) and detachment due to their intrinsic higher surface energies and weaker interactions with the support, respectively. To lower the total surface energy of the system, the nanoparticles are inclined to evolve into larger particles via agglomeration and/or Ostwald ripening, degrading the catalytic performance.^4,5^ In particular, sintering will be accelerated and worsen when the catalytic material is subjected to use at an elevated temperature. A number of methods have been proposed and/or demonstrated for mitigating the sintering process, including those that involve the use of uniform nanoparticles,^6−8^ a physical barrier to confine the nanoparticles,^9,10^ and a strong metal–support interaction.^11−13^ Among them, the use of strong metal–support interaction is most versatile, as it can be readily implemented without compromising the active sites on the metal surface. Most of the prior studies centered around noble metals and metal oxides, where the wide bandgaps of oxides tend to limit their electrical conductivity and thus compromise their catalytic performance in electrochemical applications.^14^ In comparison, carbon-based supports are advantageous in terms of electrical conductivity albeit their interactions with most noble metals are relatively weak.^15,16^ Altogether, there is a pressing need to reinforce the interaction between ultrafine Pt nanoparticles and carbon supports for the development of catalytic materials pivotal to the establishment of a clean, cost-effective, and sustainable energy infrastructure.^17^

In a recent study, we demonstrated a strategy for the *in situ* formation of carbon-supported ultrafine (1–2 nm) Pt nanoparticles by utilizing an ultrathin film of amorphous Se as the reductant.^18^ The resultant Pt/Se/C system showed superb activity and durability toward the oxygen reduction reaction (ORR) because of the strong interaction between Pt and C reinforced by the remaining Se. Specifically, we argued that Se could serve as a covalent linker to firmly anchor the Pt nanoparticles to the carbon surface. Despite the superb durability observed in an electrochemical environment, it is not clear if the Se-reinforced interaction between Pt and C can be extended to mitigate sintering at elevated temperatures, given the relatively low melting point (217 °C) of solid Se.^19−22^ The melting and then evaporation of Se are expected to weaken the interaction between Pt and the carbon surface, accelerating and worsening the sintering process. This legitimate concern calls for a systematic evaluation of the thermal stability and sinter resistance of the Pt/Se/C system.^23^

Herein, we present an *in situ* transmission electron microscopy (TEM) study of the sintering behavior of the sub-2 nm Pt nanocrystals supported on a Se-decorated carbon support at an atomic scale. The slow growth of size with temperature indicates that sintering of the Pt nanoparticles can be substantially suppressed up to about 700 °C due to the presence of Se. By probing the local atomic structure and chemical composition with high precision while monitoring the dynamic thermal behavior of the sub-2 nm Pt nanoparticles, we confirm the role played by Se in achieving superior thermal stability and sinter resistance. Our results point toward a mechanism that relies on the anchoring effect arising from the Pt–Se–C linkage.

Figure 1 shows typical *in situ* high-resolution TEM images recorded from a Pt/Se/C sample upon heating to different temperatures. At room temperature, the nanoparticles showed a uniform distribution in size (Figure 1a), with the majority of them being sub-2 nm in diameter. This size was much smaller than that (3–4 nm) of the nanoparticles typically found in a commercial Pt/C catalyst. The nanoparticles dispersed well on the Se-decorated carbon support. The specimen was then heated from 200 to 900 °C, and *in situ* images were recorded after holding at the specific temperature for 30 min. To minimize the influence of the electron beam during *in situ* heating, we quickly zoomed out to a low magnification after capturing each image.^24,25^ The *in situ* TEM images shown in Figure 1b–d display no apparent agglomeration or detachment for the nanoparticles. Meanwhile, the size distribution derived from over 200 nanoparticles indicated that the average size increased from only 1.6 to 2.2 nm when heating to 700 °C (Figure S1). Particularly, Figure 1d suggests that nanoparticles in close proximity could still be differentiated from each other. Most of the 2.2 nm Pt particles in the Pt/Se/C system remained isolated from each other rather than fusing together even at 700 °C. This remarkable sinter resistance is completely different from what has been reported in the literature for ultrafine Pt nanoparticles.^26,27^ In general, Pt nanoparticles with diameters below 2 nm are expected to undergo significant sintering, according to simple scaling models such as Herring’s law.^25,28^ However, the relatively minor size increase with temperature indicated that the sintering of Pt nanoparticles in this catalytic system was remarkably suppressed up to 700 °C. The same experiment was also repeated several times to validate the superior thermal stability and sinter resistance of the sub-2 nm Pt nanoparticles formed *in situ* on Se-decorated carbon.

**Figure 1:**
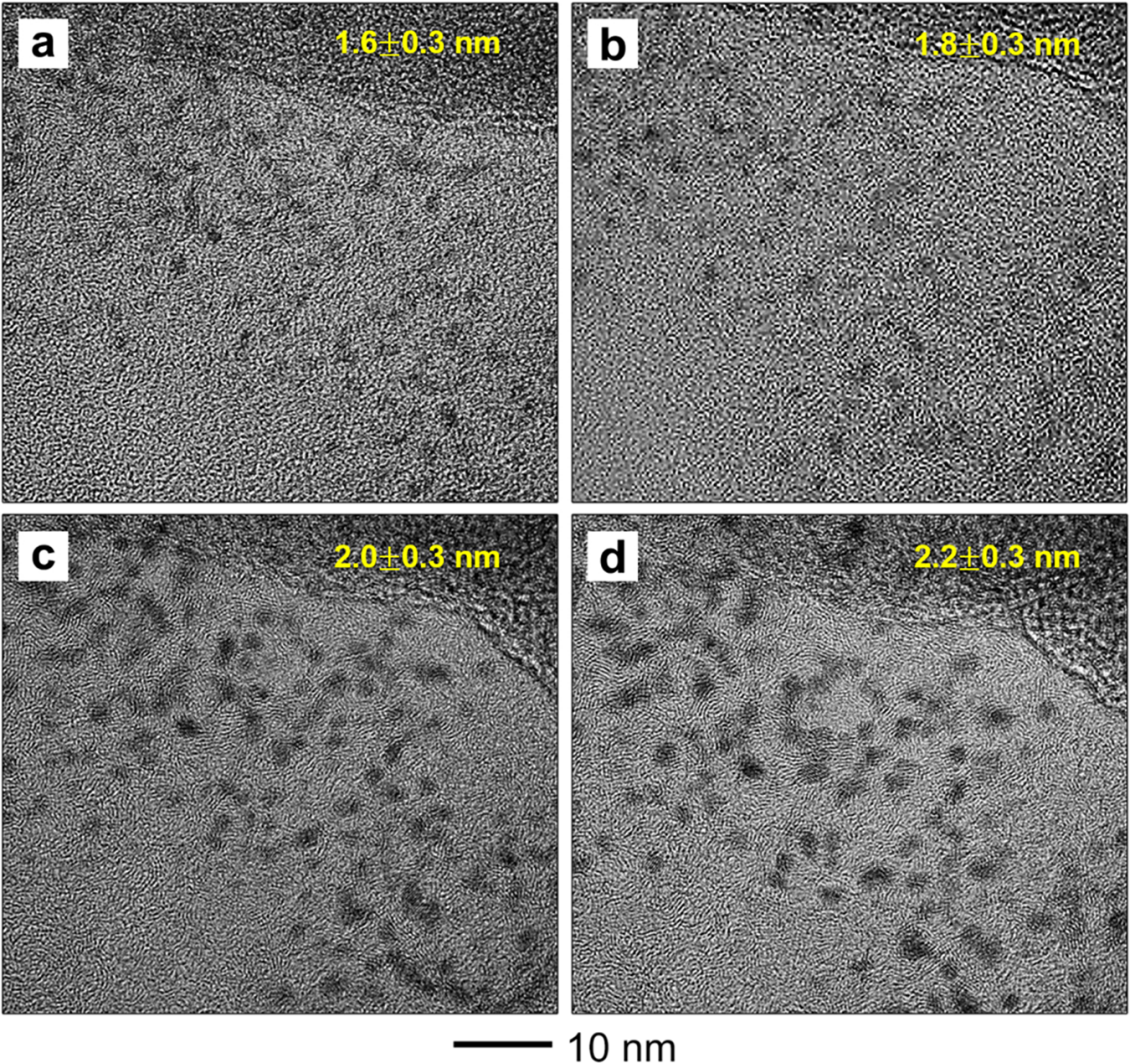
High-resolution TEM images of a Pt/Se/C sample upon heating *in situ* in an electron microscope to different temperatures and the corresponding average sizes of the Pt nanoparticles: (a) room temperature, (b) 300 °C, (c) 500 °C, and (d) 700 °C.

As we discussed before, Se could serve as a covalent linker to firmly anchor Pt nanoparticles to the carbon surface. To clearly demonstrate the effect of Pt–Se interaction on the thermal stability of this system, we also conducted a parallel *in situ* TEM study on a sample involving no Pt–Se interaction (Figure S2). The sample was prepared by directly mixing preformed 5 nm Pt nanoparticles with the carbon support, without involving Se in the sample preparation. In this case, the Pt nanoparticles started to aggregate at 500 °C due to the absence of the Pt–Se interaction. This observation provides direct evidence to support the importance of Pt–Se interaction on the superior thermal stability.

As the Pt–Se interaction is induced by using Se as a reductant for the Pt precursor, there is a significant level of skepticism regarding the actual formation of platinum selenides compounds, which is not useful for the catalytic activity. To further investigate the crystal structure and shape stability of the ultrafine nanoparticles, atomic-resolution, *in situ* STEM images were recorded from the Pt/Se/C sample annealed at 700 °C for different periods of time, and the results are presented in Figure 2a and b. The false-colored HAADF-STEM images clearly show the shape/morphology and face-centered cubic (*fcc*) crystal structure of the particle. Figure 2c shows an enlarged bright-field (BF) STEM image of the particle surface for the region indicated by the white box in Figure 2b. A structural model illustrating the periodic arrangement of Pt atoms is overlaid in the image. Gray balls were added to represent Pt atoms along the [110] zone axis. The STEM image is consistent with the projection of the Pt crystal model along the same direction, as shown in Figure 2d. The crystallinity of this particle and the *fcc* structure were also analyzed and further confirmed by the corresponding fast Fourier transform (FFT) pattern in Figure 2d. The false-colored FFT pattern from the experimental data matches the pattern Fourier-transferred from a projected atomic model of Pt along the [110] zone axis. In addition, the fringe spacings calculated from the lattice spots in the experimental FFT were 2.0 and 2.3 Å, in agreement with the separations between the {200} and {111} planes of *fcc* Pt. Taken together, it can be concluded that the crystal structure of the active Pt crystal phase and the high dispersion of the ultrafine nanoparticles were both retained even when the sample was heated to 700 °C.

**Figure 2:**
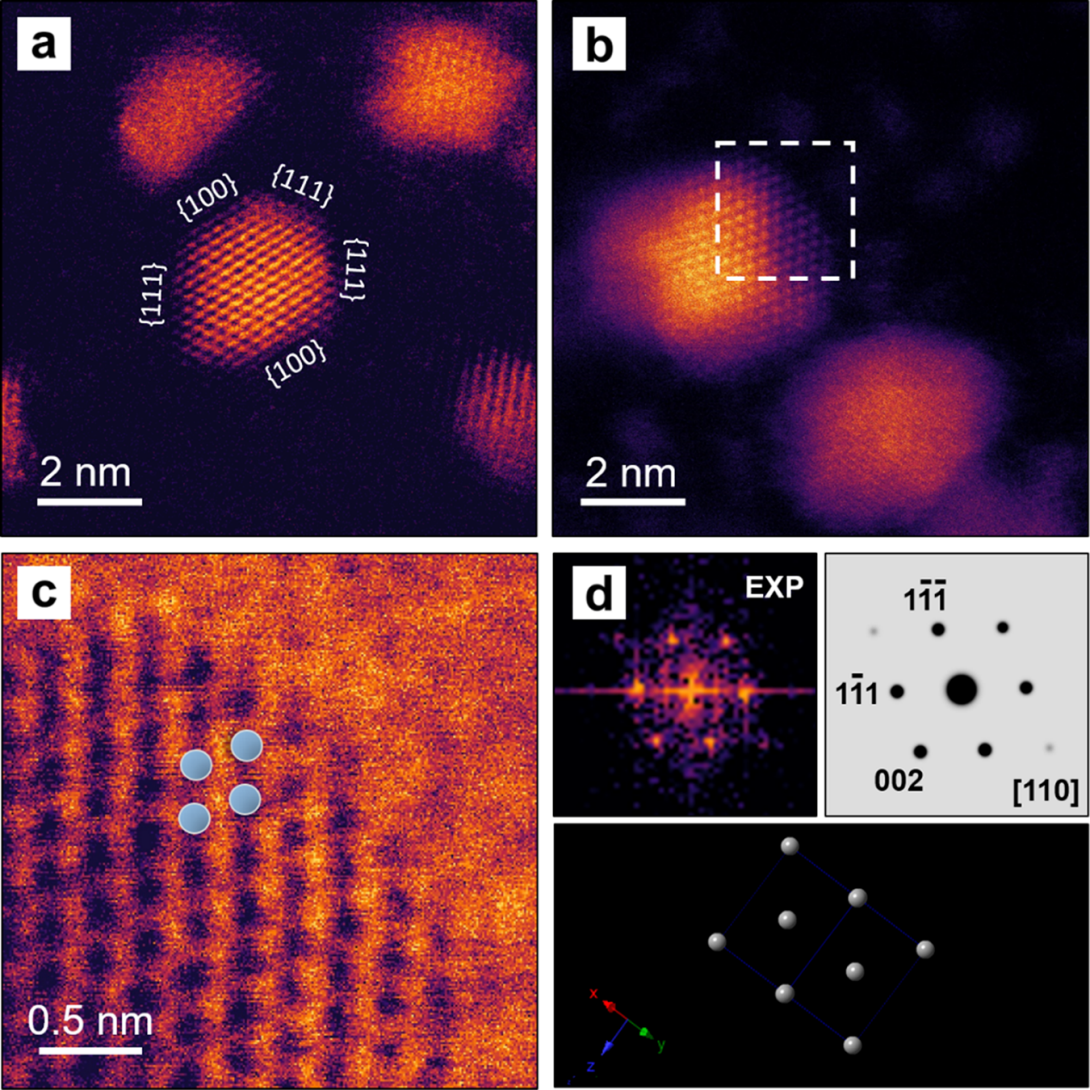
Atomic-resolution HAADF-STEM images recorded from two different regions of a Pt/Se/C sample annealed in an electron microscope at 700 °C for (a) 0.5 and (b) 1 h, respectively. (c) BF-STEM image illustrating the periodic structure of Pt taken from the surface region of a Pt nanoparticle as marked by a box in panel (b). Gray balls indicate the Pt atoms along the [110] zone axis. (d) Experimental (EXP) FFT pattern of (c) and a projected atomic model of Pt along the [110] zone axis, as well as the corresponding FFT.

When checking more nanoparticles during the heating process from 100 to 600 °C (Figure S3), the *fcc* structure was always found, without exception, clearly demonstrating the superior thermal stability of the Pt nanoparticles. In contrast, when 8 nm Pt nanoparticles were directly deposited on a TEM grid and heated to 650 °C, surface melting was observed for the outermost few atomic layers, causing the particles to change their shapes and even coalesce.^26^ On the other hand, for 18 nm Pt nanocages with walls of six atomic layers in thickness, neither of the exposed facets could be preserved above 500 °C.^29^

It is highly desirable to maintain both the shape/morphology and dispersion of the ultrafine nanoparticles on the support at elevated temperatures. To this end, there is a need to understand the sintering mechanism. In describing this thermodynamic process, the Tammann temperature (*T*_Tammann_) has been proposed as a critical parameter to determine when the Pt atoms will start to show enough mobility and thereby cause sintering.^5^*T*_Tammann_ can be roughly estimated to be half of the melting point.^30^ In the case of Pt, it has a melting point of 1768 °C.^31^ Considering the melting-point depression of nanoparticles, the decrease in particle size would significantly lower the melting point.^32,33^ Molecular dynamics simulations showed that the melting point of a 2.5 nm Pt particle was between 827 and 927 °C.^34,35^ Taken together, the theoretical *T*_Tammann_ for sub-2 nm Pt particles should be below 400 °C. Both coalescence and Ostwald ripening are expected to take place around this temperature, which contradicts our experimental observation. On the other hand, the observed sintering temperature (600–700 °C) would suggest a theoretical size of around 8 nm for the Pt nanoparticles deposited on carbon. The drastic difference between the theoretical *T*_Tammann_ and our results indicates the critical role played by the Se atoms remaining on the carbon support.

Notably, in calculating *T*_Tammann_, the metal–support interaction was neglected. Typically, the metal–support interaction is directly linked to the presence of defect sites, which govern the diffusion of metal atoms across the support surface. The migration of atoms and particles would be much slower when metal–support interactions are enhanced.^36^ However, such an enhanced metal–support interaction is often seen in oxide-based rather than carbon-based supports. Obviously, no oxide-related defects are present in the Pt/Se/C system. The sub-2 nm Pt nanoparticles in the Pt/Se/C system were formed through the galvanic reaction between a Pt(II) precursor and an amorphous Se layer predeposited on the carbon support. As such, the resultant sub-2 nm Pt nanoparticles could be linked to the residual Se and then the carbon surface.^37^ This Pt–Se interaction could dramatically affect the diffusion of the Pt nanoparticles and thus enhance their thermal stability and sinter resistance.^38^ In spite of this reasonable hypothesis, it should be noted that solid Se has a relatively low melting point at 217 °C. The actual situation of Se in the Pt/Se/C system during annealing is still unknown. It is of vital importance to reveal the nature of the Pt–Se interaction.

Both *ex situ* TEM and chemical composition analyses were applied to study the interaction between Pt and Se. We intentionally annealed the Pt/Se/C sample at 300 °C (above the melting point of Se, 217 °C) in a vacuum in the microscope. Figure 3a shows the HAADF-STEM image of the Pt/Se/C sample after being annealed for 2 h. Figure 3b shows the electron energy loss (EEL) spectra acquired from the same annealed Pt/Se/C sample. Specifically, EEL signals were collected from areas with (A1, red box) and without (A2, yellow box) Pt nanoparticles, respectively. For comparison, signals were also collected from the blank area (A3, blue box). All acquisitions were carried out under the same conditions, and the spectra were normalized to the local thickness, determined by the ratio of the low energy loss and the zero-loss peak, for further analysis. The EEL spectra suggest an apparent difference between the areas with and without Pt nanoparticles. The Se-L edge located at 1480 eV was detected from area A1, indicating that elemental Se was retained around Pt nanoparticles even after annealing at 300 °C for 2 h.^39^ Notably, the annealing temperature was above the melting point of Se by more than 80 °C. In contrast, area A2 without Pt nanoparticles showed a significant reduction in the EELS signal for the Se-L edge, indicating the presence of only a trace amount of Se in this area upon annealing at 300 °C in a vacuum. For comparison, the EEL spectrum obtained from the blank area A3 only showed background noise instead of any meaningful EEL signal, further supporting the authenticity of the Se signal from both areas A1 and A2. Altogether, the EELS data suggested that the Se atoms under or around the Pt nanoparticles were largely retained during the annealing process. If there was only Se/C without any Pt nanoparticles, such as area A2, then Se would be melted and evaporated during the annealing process.

**Figure 3:**
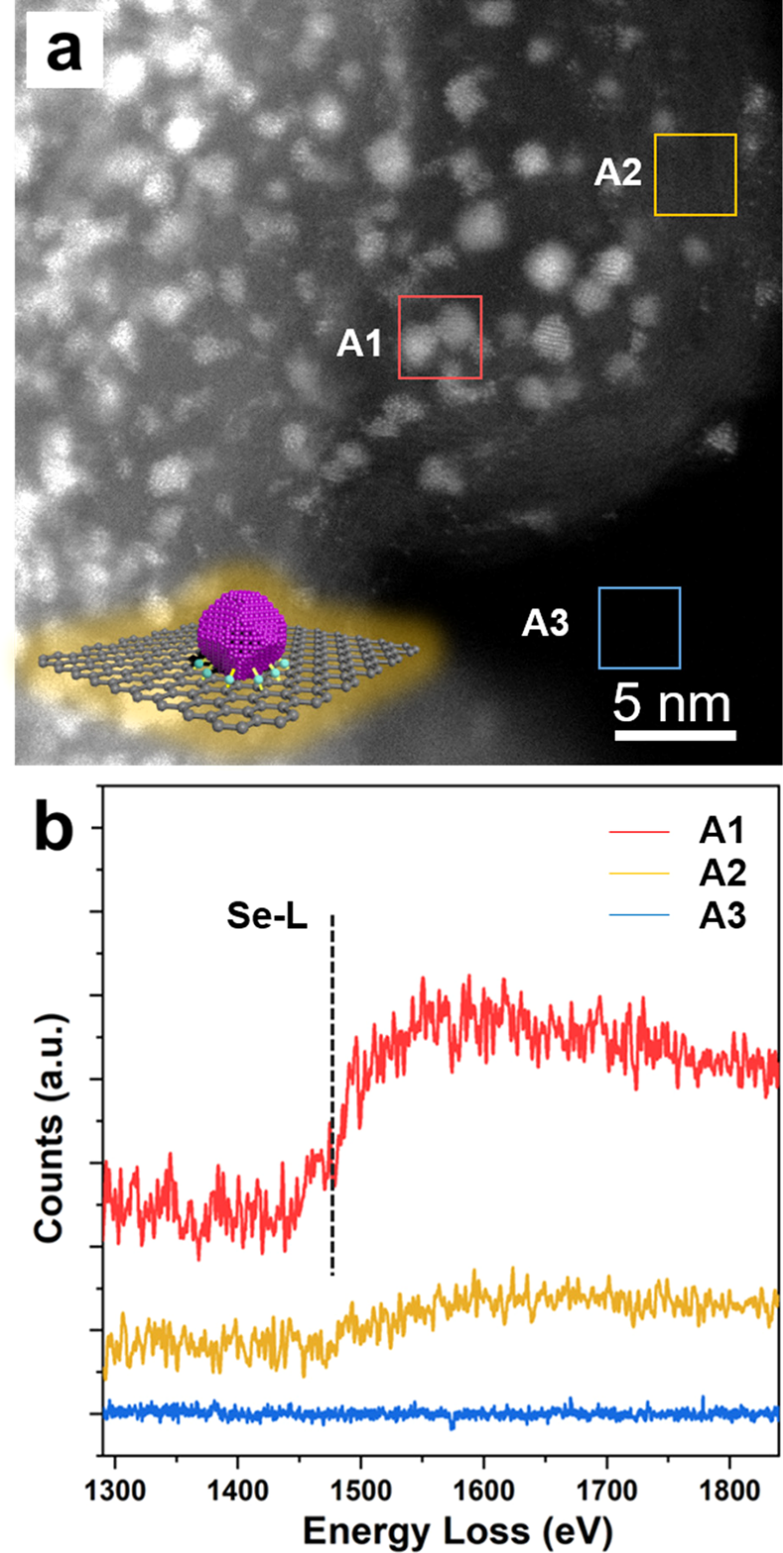
(a) HAADF-STEM image of a Pt/Se/C sample after annealing in a vacuum at 300 °C for 2 h and a schematic illustration showing how the Pt nanoparticle was anchored to the carbon surface through the Pt–Se bonding. (b) EELS spectra acquired from the different regions marked in panel (a): A1 (red box: carbon support with Pt nanoparticles), A2 (yellow box: carbon support without Pt nanoparticles), and A3 (blue box: blank area).

Given its low melting point, we argue that the Se on the carbon support could only survive the annealing process with the help of Pt–Se interaction. The EDX mapping in Figure S4 further supports our argument. The Pt and Se EDX mapping of the pristine Pt/Se/C sample clearly demonstrates a uniform distribution of Se before the annealing process. Consistent with the EEL data, EDX mapping also implies that the Pt nanoparticles interacted with the Se underneath to help preserve the elemental Se during the annealing process. From another point of view, Se could serve as a linker to firmly anchor the sub-2 nm Pt nanoparticles to the carbon surface, reducing their mobility and thus enhancing their thermal stability and sinter resistance. This anchoring effect from the Pt–Se interaction is schematically illustrated in Figure 3a.^37,40^ Since the strong interaction between Pt and Se should involve electronic interaction,^41^ selenization of the surface of the Pt nanoparticles would be expected. As such, the formation of platinum selenides, such as PtSe_2_ or Pt_5_Se_4_, on the surface is highly possible,^37^ but it is impossible to detect these selenide phases due to their extremely small domain sizes or low quantities.

To further distinguish the role of Pt–Se interaction from the formation of selenides, we calcined a Pt/Se/C sample at 250 °C outside the electron microscope but under a vacuum for 16 h. This temperature has been reported for the selenization of Pt to generate PtSe_2_.^21^ High-resolution STEM images were captured to analyze the crystal structure (Figure 4). As expected, no selenization of Pt could be observed by STEM from a plain view. The atomic images were still in agreement with the Pt models along the [110] and [112] zone axes. The lattice spacings of 0.23 and 0.20 nm in the HAADF STEM image (Figure 4a) could be assigned to the (111) and (200) planes of *fcc* Pt. No obvious PtSe_2_ or Pt_5_Se_4_ crystal spot appeared in the corresponding FFT pattern either. Although it is difficult to conclude whether the Pt/Se/C catalyst became alloyed Pt–Se or not by STEM, these images demonstrate that the amount of Se around a Pt nanoparticle was not adequate to selenize the whole nanoparticle. The XPS data from the Pt/Se/C catalyst before and after *ex situ* thermal treatment at 700 °C supported our argument (Figure S5). Essentially no change to the binding energy was observed for either the Pt 4f peak or the Se 3d peak, indicating that the electronic structures of these two elements were maintained during thermal treatment at 700 °C in a vacuum.

**Figure 4:**
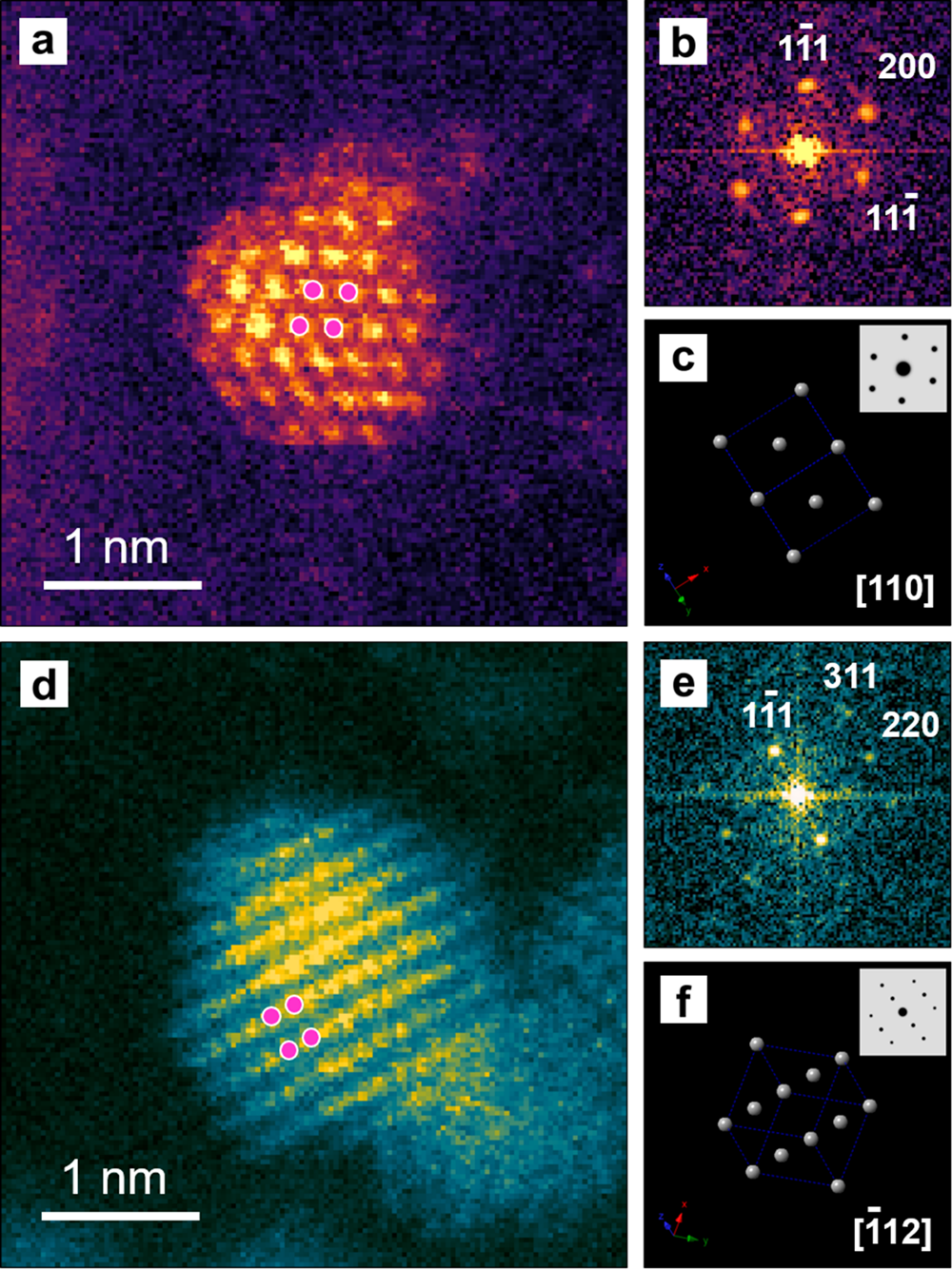
(a, d) Atomic-resolution HAADF STEM images of two sub-2 nm Pt nanoparticles after annealing in a vacuum at 250 °C for 16 h, with the pink balls representing the Pt atoms. (b, e) Corresponding FFT patterns. (c, f) Projections of Pt atoms along the [110] and [112] zone axes and the simulated FFT.

The Pt and Se would choose to interact with each other rather than form selenides due to the inadequate supply of Se. At this point, it can be concluded that the high thermal stability and sinter resistance of the Pt/Se/C system originate from the strong Pt–Se interaction, which prevents the Pt atoms, clusters, or entire particles from migrating across the surface of the carbon support.

To achieve a deeper understanding of the sintering process of this Pt/Se/C system, we further increased the annealing temperature to the theoretical melting point (*ca*. 900 °C) of 2 nm Pt nanoparticles. *In situ* low-magnification TEM images were captured to investigate the statistical behavior of the Pt nanoparticles (Figure S6). Severe sintering started to occur once the temperature reached 900 °C. We observed a “huge” nanoparticle with an irregular shape and 13 nm diameter, as well as multiple 3 nm particles. This result suggests that the sintering of the Pt nanoparticles on the carbon support proceeded through coalescence rather than Ostwald ripening at such a high temperature. In general, Ostwald ripening is driven by the reduction in surface energy, and small Pt nanoparticles are dissolved and redeposited onto larger ones.^42^ In contrast, coalescence gives rise to a decrease in particle number density.^3^ The size distribution at 700 °C also delivered the same message. If Ostwald ripening was involved, the small end of the particle size distribution should have shifted more significantly relative to the pristine sample.^43^ Since small nanoparticles still existed while the number of particles dropped dramatically, we argue that coalescence was mainly responsible for the sintering observed at 900 °C.

In conclusion, we have investigated the mechanism responsible for the extraordinary thermal stability and sinter resistance of sub-2 nm Pt nanoparticles on Se-decorated carbon by performing *in situ* TEM/STEM analyses. The average size of the Pt nanoparticles only increased from 1.6 to 2.2 nm when heating up to 700 °C. Both the {111} and {100} facets on the surface were well retained even at 700 °C. Further elevating the temperature to 900 °C, the theoretical melting point of 2 nm Pt nanoparticles, led to coalescence and thus sintering of the nanoparticles. By systematically analyzing the chemical evolution of the Pt/Se/C sample, we could attribute the extraordinary thermal stability to the anchoring effect arising from the strong Pt–Se interaction. Different from the uniform distribution of Se in the pristine sample, the preferential concentration of Se under or around Pt nanoparticles strongly supports our argument. The study presented here not only sheds light on the mechanism responsible for sinter resistance but also paves the way for the development of high-performance industrial catalysts.

## Supporting Information Available

Additional experimental details, materials, and methods of synthesis; TEM experimental setup and analytical methods; size distributions of the Pt nanoparticles after annealing at different temperatures; in situ heating TEM images of a sample involving no Pt–Se interaction at different temperatures; atomic-resolution TEM images of the sub-2 nm Pt nanoparticles after annealing at different temperatures; EDX mapping of the Pt/Se/C sample; XPS spectra of the Pt/Se/C sample before and after thermal treatment at 700 °C; and in situ TEM image of the Pt/Se/C sample at 900 °C and the possible sintering mechanisms (PDF)
