# Supplementary material for: Extraordinary Thermal Stability and Sinter Resistance of Sub-2 nm Platinum Nanoparticles Anchored to a Carbon Support by Selenium
Source: Nano Lett. 2024 Jan 16;24(4):1392–8. doi: 10.1021/acs.nanolett.3c04601 (PMC10835721; doi:10.1021/acs.nanolett.3c04601)
Supplement: Supplementary file 1 — nl3c04601_si_001.pdf [file nl3c04601_si_001.pdf]

Supporting Information

Extraordinary Thermal Stability and Sinter Resistance of Sub-2-nm Platinum Nanoparticles Anchored to a Carbon Support by Selenium

Zitao Chen,[†,‡] Haoyan Cheng,[†] Zhenming Cao,[†] Jiawei Zhu,[†] Thomas Blum,[⊥] Qinyuan Zhang,[‡] Miaofang Chi,[⊥,*] and Younan Xia[†,§,*]

[†]The Wallace H. Coulter Department of Biomedical Engineering, Georgia Institute of Technology and Emory University, Atlanta, Georgia 30332, United States

[‡]State Key Laboratory of Luminescent Materials and Devices, South China University of Technology, Guangzhou 510641, China

[⊥]Center for Nanophase Materials Sciences, Oak Ridge National Laboratory, Oak Ridge, Tennessee 37831, United States

[§]School of Chemistry and Biochemistry, Georgia Institute of Technology, Atlanta, Georgia 30332, United States

*Address correspondence to chim@ornl.gov and younan.xia@bme.gatech.edu

Methods

Chemicals and materials. Selenous acid (H_2SeO_3, >99.99%), hydrazine monohydrate ($N_2H_4 \cdot H_2O$, >98%), poly(vinyl pyrrolidone) (PVP with MW≈55,000), potassium platinum(II) chloride (K_2PtCl_4, >99.99%), platinum(II)-ammonium chloride ($(NH_4)_2PtCl_4$, 99%), sodium platinum(IV) hexachloride hexahydrate ($Na_2PtCl_6 \cdot 6H_2O$, 98%), and chloroplatinic acid hexahydrate ($H_2PtCl_6 \cdot xH_2O$, >99.99%) were all obtained from Sigma-Aldrich (St. Louis, MO) and used as received. The deionized (DI) water (18.2 MΩ·cm) obtained by purification through a Milli-Q system (Millipore, USA) was used throughout the experiments.

Standard procedure for the *in situ* growth of Pt nanoparticles on carbon support. The method was the same as the standard protocol we reported before.[1] Typically, 1 mL of the aqueous suspension of Se/C (1 mg/mL, obtained after thermal treatment at 450 °C) was diluted to 10 mL by adding water and then heated to 70 °C under magnetic stirring. Once the temperature was stable, 150 μL of an aqueous solution of K_2PtCl_4 (10 mM) was added in one shot and the reaction was allowed to continue for 2 h under magnetic stirring. The solid product was collected by centrifugation, washed three times with water, and dispersed in 1 mL of water for further use. The final product was denoted standard Pt/Se/C.

Procedure for physically mixing Pt nanoparticles with Se-decorated carbon powders. A suspension of 5-nm Pt nanoparticles was mixed with another aqueous suspension of Se/C (20 mL). The mixture was then sonicated for 1 h. The solid product was collected by centrifugation, washed three times with water, and dispersed in 1 mL of water for further use.

***In situ* TEM/STEM analysis.** The TEM/STEM images were taken using an aberration-corrected FEI Titan S 80-300 STEM/TEM microscope equipped with a Gatan OneView camera at an accelerating voltage of 300 kV at Oak Ridge National Laboratory (ORNL). An aqueous suspension of the as-prepared Pt/Se/C sample was drop-cast onto the Aduro thermal device and then allowed to dry under ambient conditions. Protochips Aduro heating holder was used for the *in situ* heating experiments to control the temperature.[2] The specimen was heated from room temperature to 900 °C at a rate of 50 °C s^{-1} and held for 30 min at each temperature. Ten areas containing multiple Pt nanoparticles were tracked during the annealing process. The specimen was exposed to the electron

beam only during data acquisition. The same experiment was repeated once to confirm the evolution of size and crystal structure in the Pt nanoparticles.

***Ex situ* STEM and chemical composition analysis.** The *ex-situ* STEM and electron energy loss (EEL) analyses were carried out using the same heating holder to heat the sample inside the Fischione Instruments plasma cleaner and then transferred to the same Titan microscope. Energy-dispersive X-ray elemental mapping was acquired using an aberration-corrected JEOL NEOARM STEM/TEM microscope operated at 200 kV with dual large-angle EDX-SDD detectors at ORNL.

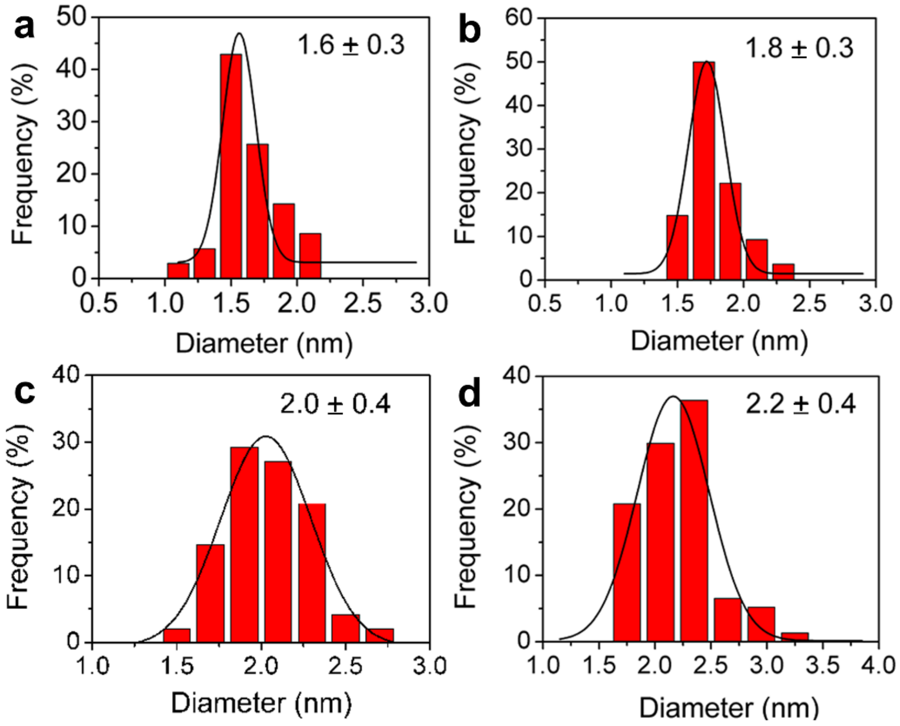

Figure S1. Size distributions of the Pt nanoparticles in a sample after annealing in the microscope at different temperatures for 30 min: (a) room temperature, (b) 300, (c) 500, and (d) 700 ºC.

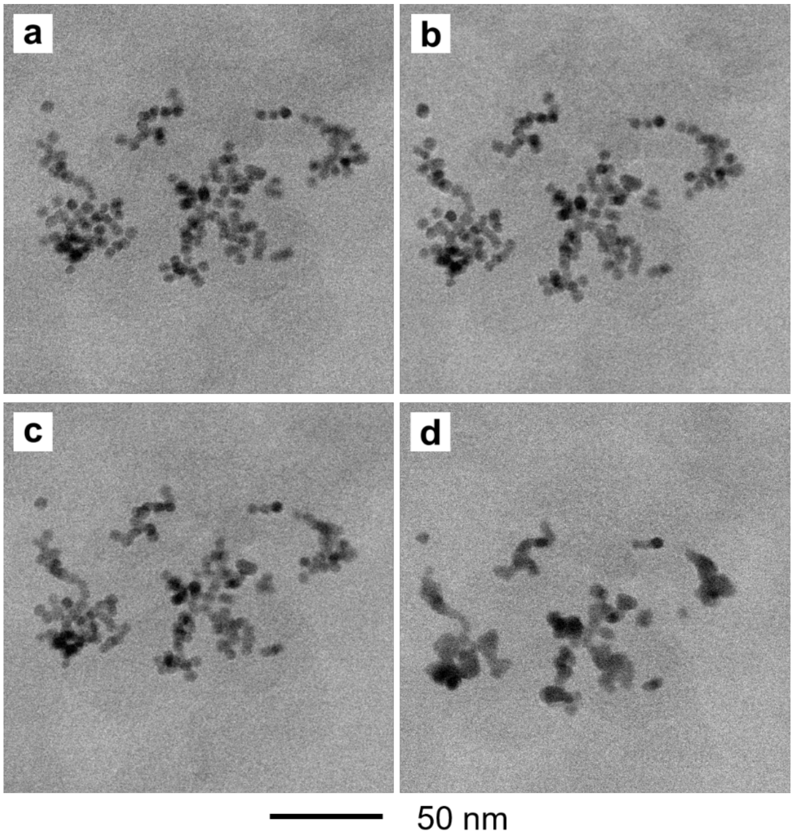

Figure S2. Sequential *in-situ* TEM images of a sample involving no Pt-Se interaction, which was prepared by physically mixing preformed 5-nm Pt nanoparticles with carbon powders, after annealing at different temperatures: (a) room temperature, (b) 100, (c) 300, and (d) 500 ^{o}C.

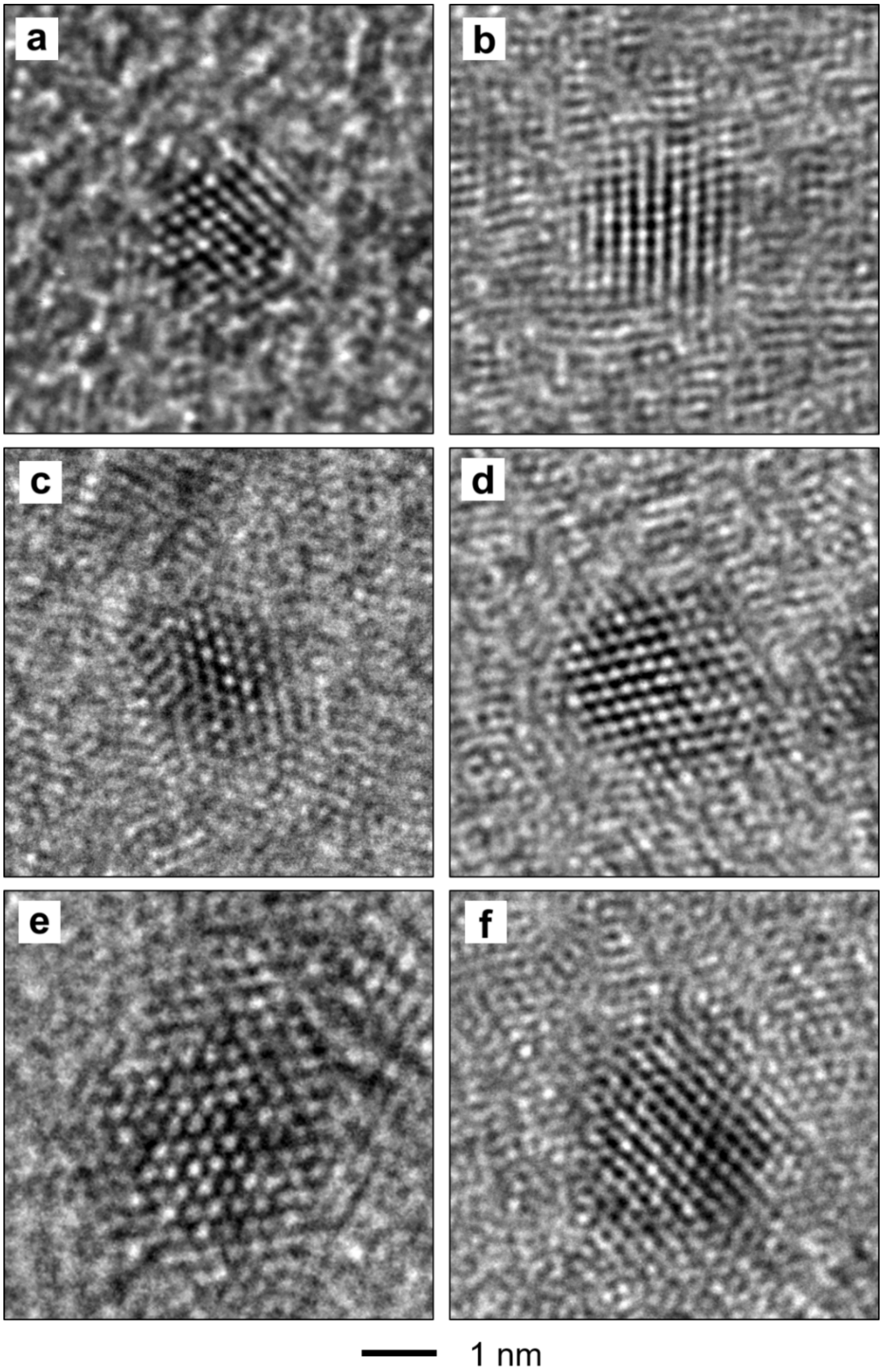

Figure S3. Atomic-resolution TEM images of distinct sub-2-nm Pt nanoparticles from samples after annealing at different temperatures: (a) 100, (b) 200, (c) 300, (d) 400, (e) 500, and (f) 600 ºC

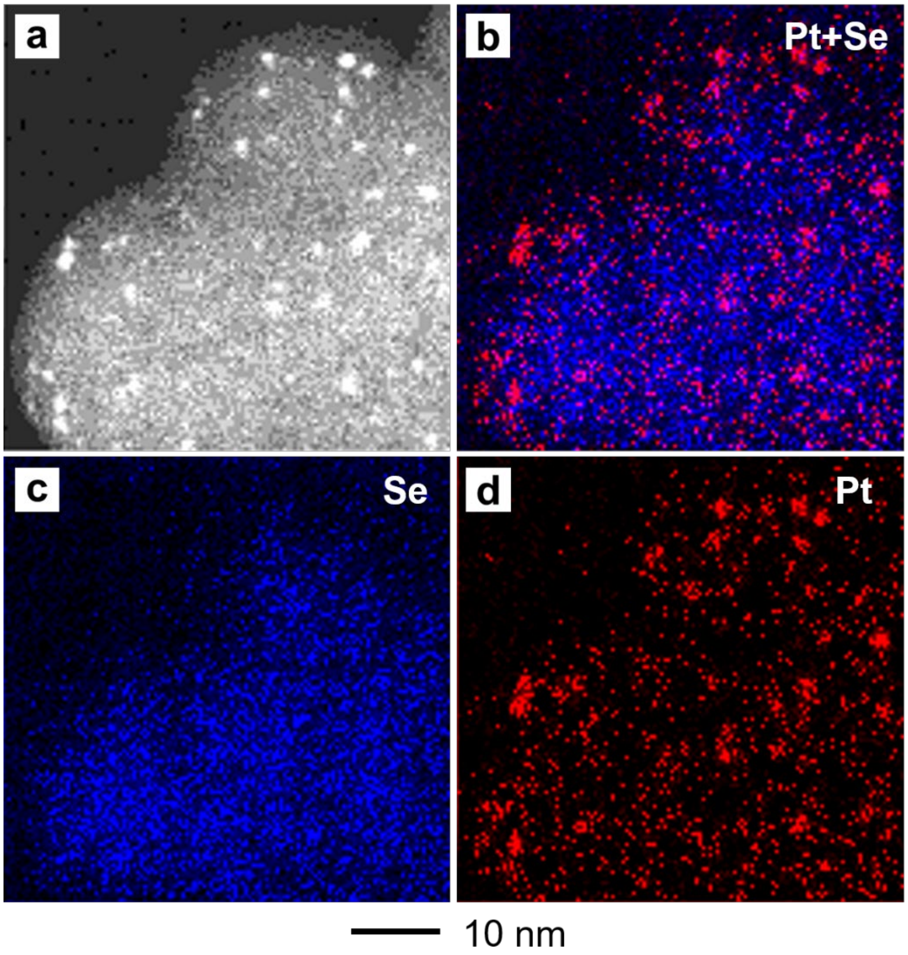

Figure S4. Energy-dispersive X-ray spectroscopy (EDX) mapping of a Pt/Se/C sample (Se, blue, Pt, red) without thermal annealing.

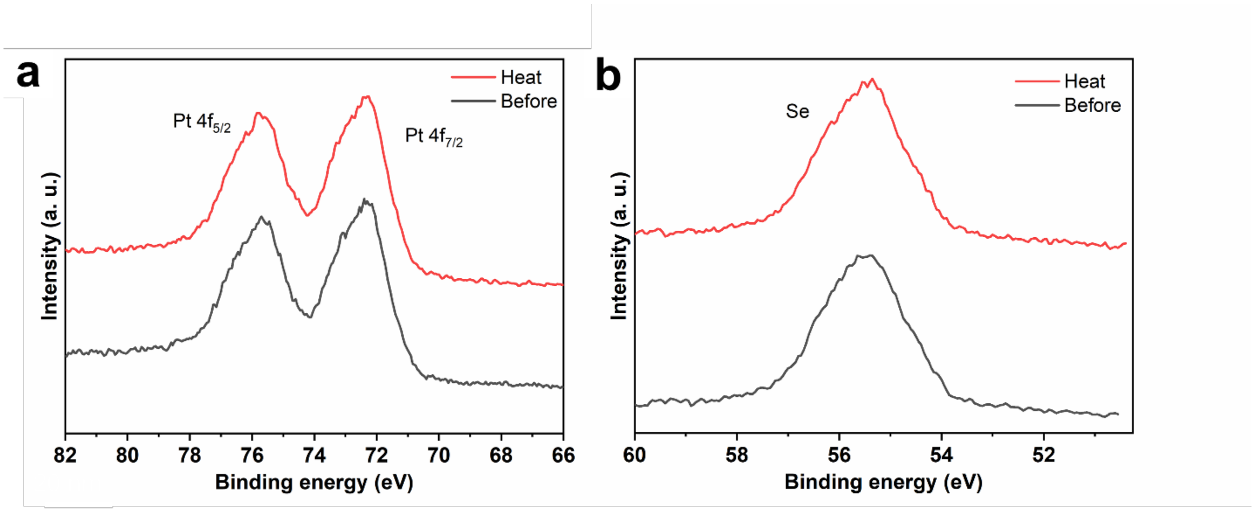

Figure S5. (a) Pt 4f XPS spectra and (b) Se 3d XPS spectra recorded from Pt/Se/C before and after *ex-situ* thermal treatment at 700 °C in vacuum for 30 min, respectively.

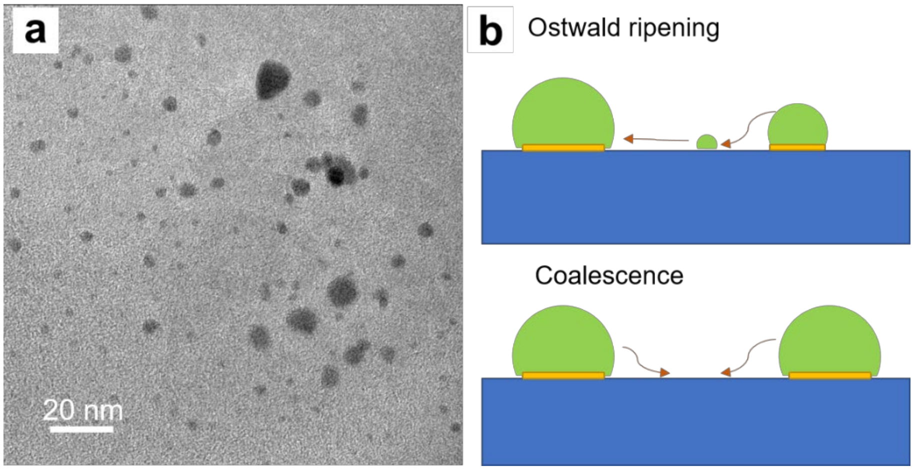

Figure S6. *In situ* low-magnification TEM image of the Pt/Se/C sample annealed at 900 ºC for 30 min and the two possible sintering mechanisms.

References

(1) Cheng, H.; Cao, Z.; Chen, Z.; Zhao, M.; Xie, M.; Lyu, Z.; Zhu Z.; Chi M.; Xia, Y. Catalytic System based on sub-2 nm Pt particles and its extraordinary activity and durability for oxygen reduction. *Nano lett.* **2019**, *19*, 4997-5002.

(2) Allard, L. F.; Bigelow, W. C.; Jose-Yacaman, M.; Nackashi, D. P.; Damiano, J.; Mick, S. E. A new MEMS-based system for ultra-high-resolution imaging at elevated temperatures. *Microsc. Res. Techniq.* **2009**, *72*, 208-215.
